# Pattern-Recognition Receptors and Gastric Cancer

**DOI:** 10.3389/fimmu.2014.00336

**Published:** 2014-07-22

**Authors:** Natalia Castaño-Rodríguez, Nadeem O. Kaakoush, Hazel M. Mitchell

**Affiliations:** ^1^School of Biotechnology and Biomolecular Sciences, The University of New South Wales, Sydney, NSW, Australia

**Keywords:** stomach neoplasm, *Helicobacter pylori*, inflammation, pattern-recognition receptors, Toll-like receptors, NOD-like receptors, genetic polymorphism, therapeutics

## Abstract

Chronic inflammation has been associated with an increased risk of several human malignancies, a classic example being gastric adenocarcinoma (GC). Development of GC is known to result from infection of the gastric mucosa by *Helicobacter pylori*, which initially induces acute inflammation and, in a subset of patients, progresses over time to chronic inflammation, gastric atrophy, intestinal metaplasia, dysplasia, and finally intestinal-type GC. Germ-line encoded receptors known as pattern-recognition receptors (PRRs) are critical for generating mature pro-inflammatory cytokines that are crucial for both Th1 and Th2 responses. Given that *H. pylori* is initially targeted by PRRs, it is conceivable that dysfunction within genes of this arm of the immune system could modulate the host response against *H. pylori* infection, and subsequently influence the emergence of GC. Current evidence suggests that Toll-like receptors (TLRs) (TLR2, TLR3, TLR4, TLR5, and TLR9), nucleotide-binding oligomerization domain (NOD)-like receptors (NLRs) (NOD1, NOD2, and NLRP3), a C-type lectin receptor (DC-SIGN), and retinoic acid-inducible gene (RIG)-I-like receptors (RIG-I and MDA-5), are involved in both the recognition of *H. pylori* and gastric carcinogenesis. In addition, polymorphisms in genes involved in the TLR (*TLR1, TLR2, TLR4, TLR5, TLR9*, and *CD14*) and NLR (*NOD1, NOD2, NLRP3, NLRP12, NLRX1, CASP1, ASC*, and *CARD8*) signaling pathways have been shown to modulate the risk of *H. pylori* infection, gastric precancerous lesions, and/or GC. Further, the modulation of PRRs has been suggested to suppress *H. pylori*-induced inflammation and enhance GC cell apoptosis, highlighting their potential relevance in GC therapeutics. In this review, we present current advances in our understanding of the role of the TLR and NLR signaling pathways in the pathogenesis of GC, address the involvement of other recently identified PRRs in GC, and discuss the potential implications of PRRs in GC immunotherapy.

## Introduction

Of the three main types of stomach cancer, gastric adenocarcinoma (GC), non-Hodgkin’s lymphoma, and gastrointestinal stromal tumors, approximately 95% are GC, which remains one of the most commonly diagnosed cancers in the world (1). In 2012, stomach cancer was the fifth most common cancer worldwide, with 952,000 new cases diagnosed, accounting for 6.8% of the total cancer cases (1). Furthermore, it is the third leading cause of cancer-related deaths worldwide, accounting for 8.8% of total deaths from cancer, with 5-year relative survival rates lower than 30%, except in Japan where mass screening has been undertaken for several years (2).

**Figure 1:**
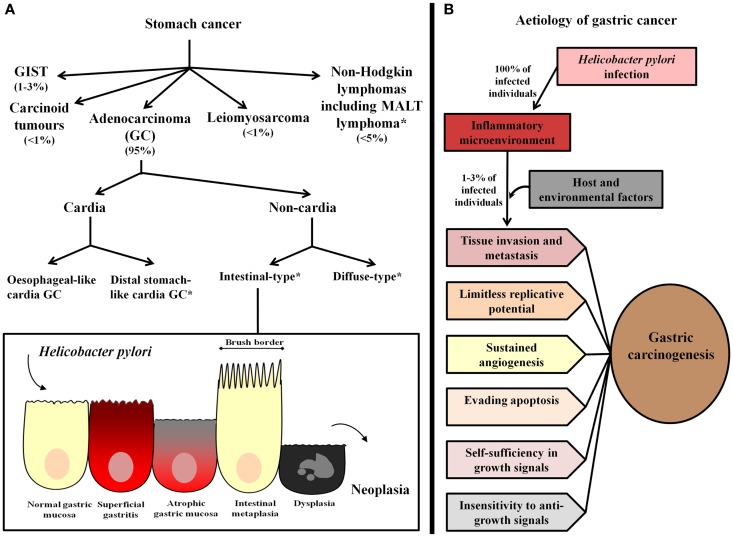
**Gastric cancer classification and etiology**. **(A)** Stomach cancer comprises gastric adenocarcinoma (GC), non-Hodgkin lymphomas, including mucosa-associated lymphoid tissue (MALT) lymphoma, and the rare gastrointestinal stromal tumors (GIST), leiomyosarcoma, and carcinoid tumors. The most common type, GC, has been classified as cardia and non-cardia GC according to anatomical location. Cardia GC is divided into two different etiological entities, esophageal-like cardia GC, which is associated with gastro-esophageal reflux, smoking, and diet, and is frequent in areas with a low risk of GC and distal stomach-like cardia GC, which is associated with the presence of *H. pylori* and gastric atrophy, and is the most frequent cardia GC variant in areas with a high risk of GC. Non-cardia GC is further subdivided into two histological variants called intestinal-type and diffuse-type GC. Intestinal-type GC, according to the widely accepted Correa’s cascade (7), is a biological continuum that commences as chronic gastritis and progresses to atrophic gastritis, intestinal metaplasia, dysplasia, and finally, GC. *Stomach cancer subtypes that have been associated with *Helicobacter pylori* infection. (**B**) *H. pylori* infection causes chronic inflammation of the gastric mucosa of all infected individuals, and in combination with host and environmental factor, leads to the development of GC in a subset of infected individuals (1–3%). In these subjects, inflammation represents the seventh hallmark of cancer and an enabling characteristic that facilitates the acquisition of the other established hallmarks that collectively dictate malignant growth (tissue invasion/metastasis, limitless replicative potential, sustained angiogenesis, evasion of programed-cell death (apoptosis), self-sufficiency in growth signals, and insensitivity to growth-inhibitory signals) (8, 9).

Gastric cancer is a heterogeneous pathology with respect to anatomical location and histological subtypes (Figure 1A). In relation to location, GC may occur in the cardia or non-cardia region of the stomach. Cardia GC has been associated with gastro-esophageal reflux, *Helicobacter pylori* infection, and atrophic gastritis, male gender, smoking, and diet (3). Epidemiological studies assessing the worldwide incidence of GC by anatomical location have shown an increase in the incidence of cardia GC, however, in high GC risk areas, non-cardia GC remains the most frequent pathology (4). Further, even though cardia and non-cardia GC have been considered etiologically different phenomena, it has been demonstrated that cancer of the cardia among individuals from areas with a high risk of GC represents a subset of cardia GC that is associated with *H. pylori*-related atrophic gastritis and resembles non-cardia GC pathogenesis (5, 6).

According to the Lauren Classification, non-cardia GC has been further subdivided into the two histological variants intestinal-type and diffuse-type. Intestinal-type GC is characterized by the formation of gland-like structures, distal stomach localization, and a predilection for older individuals. It is also more frequent in males (2:1 ratio) and in subjects of lower socioeconomic status (10). This type of GC is often preceded by a precancerous phase that starts with the transition of normal mucosa into multifocal atrophic gastritis. This initial histological alteration is followed by intestinal metaplasia, dysplasia, and finally adenocarcinoma (11). On the other hand, diffuse-type GC is poorly differentiated, affects younger individuals, and has been highly associated with genetic susceptibility (the variant hereditary diffuse GC, which is associated with germ-line mutations in *CDH1*, a gene encoding E-cadherin) (12, 13). Additionally, it is not associated with the formation of precancerous lesions and has been found to affect the entire surface of the stomach. This type of GC is present equally between the two sexes and is associated with a worse prognosis in comparison to intestinal-type GC (10, 12).

Most GC cases are sporadic and arise due to the combination of a permissive environment interacting with a susceptible host. Several factors that contribute to the development of GC have been identified; these include bacterial (*H. pylori*), host, and environmental factors (12).

*Helicobacter pylori* is a Gram-negative bacterium that infects nearly 50% of the human population (14). In the gastric mucosa, the majority of *Helicobacter pylori* are found within the mucus layer but they can also be attached to epithelial cells leading to the maintenance, spread, and severity of the infection (15). *H. pylori* infection has been associated with the development of a range of diseases, including peptic ulcer disease (10%), non-cardia GC (1–3%), and gastric mucosa-associated lymphoid tissue (MALT) lymphoma (<0.1%) (14, 16–18). Furthermore, this bacterium has been associated with three distinct phenotypes in the infected host: (1) a corpus-predominant gastritis, which has the potential to lead to atrophic gastritis, hypochlorhydria, and to the development of GC; (2) a duodenal ulcer phenotype in which an antrum-predominant gastritis leads to increased gastric acid secretion; and (3) a benign phenotype in which the bacterial infection causes a mild mixed gastritis that has a minor effect on gastric acid production (19).

*Helicobacter pylori* infection is transmitted by direct human-to-human transmission, via either the oral–oral route, fecal–oral route, or both (14). *H. pylori* is acquired early in life, the majority of individuals being infected before the age of 10 years with close family members being a common source of infection (20–22). It has been postulated that early acquisition of infection might be associated with the broad pathological spectrum associated with *H. pylori* infection and the highly persistent GC incidence rates in genetically susceptible populations who have migrated to developed countries. In the absence of antibiotic therapy, *H. pylori* infection generally persists for life (23).

Natural colonization by *H. pylori* is restricted to humans, primates, and domestic animals such as cats (23–25). *H. pylori* is considered to be the dominant microorganism in the human stomach as the majority of bacteria cannot survive in the low gastric pH (26). Several other factors make the human stomach an unfavorable environment for bacterial colonization including peristalsis, poor nutrient availability, and host innate and adaptive immunity (23). The ability of *H. pylori* to survive and colonize the stomach relates to a number of mechanisms. Most importantly *H. pylori*, unlike other bacteria, produces large amounts of the enzyme urease, which hydrolyzes urea to ammonia, which subsequently interacts with hydrogen ions in the stomach to form ammonium (27, 28). In addition, *H. pylori* is able to regulate gene expression in response to changes in pH (29). Further, *H. pylori* expresses multiple paralogous outer membrane proteins, including the blood-group antigen-binding adhesin (BabA), the sialic-acid binding adhesin (SabA), and the outer inflammatory protein (OipA), which appear to bind to receptors on the surface of gastric epithelial cells, which reduces the rate of bacterial elimination as a result of peristalsis (30, 31). *H. pylori* counteracts the lack of nutrients by inducing tissue inflammation and using specific systems that facilitate the transport and uptake of nutritional resources (23). In addition, *H. pylori* has been reported to produce antibacterial peptides that might decrease competition from other microorganisms (32).

Further, a number of other factors have been shown to help *H. pylori* evade the host immune system. For example, the vacuolating cytotoxin (VacA) produced by some strains of *H. pylori* has been shown to inhibit T-cell proliferation as well as antigen presentation by B cells and to alter the normal functions of CD8^+^ T cells, mast cells, and macrophages (33–36). In addition, gamma-glutamyl transpeptidase, another immunosuppressive factor of *H. pylori*, has been associated with inhibition of T-cell proliferation by induction of a cell cycle arrest in the G_1_ phase (37). Furthermore, *H. pylori* has been shown to use arginase to down-regulate the production of inducible nitric oxide synthase by macrophages (38).

The fact that more than one *H. pylori* strain can colonize the gastric mucosa provides the opportunity for *H. pylori* to acquire new genetic sequences and to undergo recombination events (23). One of the most remarkable differences among *H. pylori* strains is the presence or absence of a 40-kb DNA insertion element known as the cytotoxin-associated gene pathogenicity island (*cag* PAI) (39). This region contains between 27 and 31 genes flanked by 31-bp repeats and encodes the most widely investigated *H. pylori* virulence factor, the cytotoxin-associated antigen A (CagA) (40, 41). *H. pylori* strains expressing CagA represent 60–70% of Western strains and approximately 100% of East Asian strains (39, 42). CagA is a 120- to 140-kDa protein that is translocated into host cells through a type IV secretion system following attachment to gastric epithelial cells (43). Following translocation, CagA is tyrosine phosphorylated at the EPIYA (glutamate–proline–isoleucine–tyrosine–alanine) motifs by members of the host cell kinase families known as proto-oncogene proteins Abl and Src (18). In Western populations strains, EPIYA-A, EPIYA-B, and varying numbers of EPIYA-C motifs have been reported, while in *H. pylori* strains from East Asian populations, EPIYA-A and EPIYA-B with EPIYA-D motifs, are found (44). Both phosphorylated and non-phosphorylated CagA result in alterations in the gastric epithelium including: (1) the activation of the protein tyrosine phosphatase, non-receptor type 11 (SHP-2), (2) alterations in cell scattering and proliferation, (3) alterations in cell structure and cell motility, (4) perturbation of epithelial cell differentiation and polarity, (5) alteration of tight junctions, and (6) aberrant activation of β-catenin (45–47). Furthermore, numerous studies have shown that *cag* PAI-positive *H. pylori* strains are associated with an increased risk of gastric diseases including peptic ulcer disease, premalignant gastric lesions and GC (48–51). Further details of the interplay between *H. pylori* virulence factors and gastric epithelial cells and GC, can be found in an excellent review by Posselt et al. (44).

In the last two decades, a large number of epidemiological studies have established the association between *H. pylori* and the subsequent risk of developing both intestinal-type and diffuse-type GC (52–57). This finding has been consistent among different populations. For example, in the study by Parsonnet et al. (57), conducted in Caucasian, African-American, and Asian individuals, subjects infected with *H. pylori* who had antibodies against CagA were shown to be more likely than uninfected subjects to develop both intestinal-type and diffuse-type GC (OR: 5.1, 95% CI: 2.1–12.2 and OR: 10.1, 95% CI: 2.2–47.4, respectively). Consistently, a further study conducted in a Japanese population showed that, although the association was stronger in cases with intestinal-type GC (OR: 3.2, 95% CI: 1.8–5.8), there was also a positive association between *H. pylori* infection and diffuse-type GC (OR: 3.0, 95% CI: 1.0–8.8) (53). Further, a study conducted in a Spanish population showed no differences in *H. pylori* infection between the two GC histological subtypes (58). Similarly, a recent study in German individuals showed that *H. pylori* prevalence was comparable in patients with intestinal-type (82.1%) and diffuse-type (77.9%) GC (59).

Interestingly, more recent studies, assessing *H. pylori* infection through Western blot (CagA) for the detection of past infection, have shown an unprecedented association between *H. pylori* and GC that can be explained by a reduction of the misclassification that might take place when samples are analyzed with the enzyme-linked immunosorbent assay (ELISA) alone (60, 61). For example, Ekstrome et al. (60) conducted a population-based study, comprising 298 GC patients and 244 controls, in which the OR for *H. pylori* infection among non-cardia GC was 21.0 (95% CI: 8.3–53.4). Further, Siman et al. (61) showed that *H. pylori* significantly increased the risk of non-cardia GC showing an OR of 17.8 (95% CI: 4.2–74.8).

While *H. pylori* infection has been established as the most important risk factor for GC and was classified as a class 1 carcinogen by the World Health Organization in 1994, the etiology of GC also involves host and environmental factors. This is evidenced by the fact that only 1–3% of *H. pylori*-infected patients develop GC, and that progression to GC in some subjects occurs even after eradication of the bacterium (18).

Given that *H. pylori* is initially targeted by germ-line encoded receptors known as pattern-recognition receptors (PRRs), it is conceivable that dysfunction within genes of this arm of the immune system would affect the magnitude and direction of the host inflammatory response against the infection, resulting in an increased risk of GC development. Recent studies clearly show that PRRs are critical for generating mature pro-inflammatory cytokines that are crucial for both Th1 and Th2 responses during *H. pylori* infection, and these immune responses have been directly associated with gastric immunopathology. In this review, we present current advances in the understanding of the role of PRRs, mainly the Toll-like receptor (TLR) and nucleotide-binding oligomerization domain (NOD)-like receptor (NLR) signaling pathways, in the pathogenesis of GC, and discuss future directions for continued research in this area. In the first section, we highlight the relevance of inflammation in GC. In subsequent sections, we address new developments in the TLR and NLR signaling pathways in GC, the role of other PRRs in GC, and the new frontier of therapeutic application of these concepts.

## Inflammation in Gastric Cancer

It is well established that most cancer cell genotypes are the manifestation of six essential alterations in cell physiology that collectively dictate malignant growth: (1) self-sufficiency in growth signals, (2) insensitivity to growth-inhibitory signals, (3) evasion of programed-cell death (apoptosis), (4) limitless replicative potential, (5) sustained angiogenesis, and (6) tissue invasion/metastasis (8). Recently, inflammation has been considered the seventh hallmark of cancer and an enabling characteristic that facilitates the acquisition of the other hallmarks (Figure 1B). Inflammation initiated by innate immune cells, mainly macrophage subtypes, mast cells, myeloid progenitors, and neutrophils (62–65), designed to fight infections and heal wounds, can instead result in unintentional support of multiple cancer hallmark functions, thereby manifesting the widely accepted tumor-promoting consequences of inflammatory responses (9). In addition, active evasion by cancer cells from attack and elimination by immune cells, mainly CD8+ cytotoxic T lymphocytes, CD4+ Type 1 helper T cells, and natural killer (NK) cells, highlights the dual role of an immune system that both antagonizes and promotes cancer development and progression (9).

In the context of tumor enhancement, it has been proposed that once inflammation is initiated, tissue integrity is compromised leading to the multistage process of carcinogenesis by altering targets and pathways that are pivotal for normal tissue homeostasis (66). The mechanisms that are connected to these alterations include production of mutagenic reactive oxygen and nitrogen species as well as synthesis of cytokines and growth factors that favor tumor cell growth (67). In addition, inflammation provides a source of other bioactive molecules to the tumor microenvironment, including survival factors that limit cell death, pro-angiogenic factors, extracellular matrix-modifying enzymes that facilitate angiogenesis, invasion, and metastasis, and inductive signals that lead to activation of the epithelial–mesenchymal transition (a developmental regulatory program that enables epithelial cells to invade, resist apoptosis, and disseminate) (9). Interestingly, inflammation can be considered a “perigenetic alteration” of cancer cells because it may promote growth, expansion, and invasion of tumors even without the involvement of further genetic mutations or epigenetic alterations (68).

In 1988, Correa proposed a human model of intestinal-type gastric carcinogenesis (7). The model hypothesized a sequence of events progressing from acute inflammation to chronic inflammation, to atrophy, to intestinal metaplasia, to dysplasia, to carcinoma *in situ*, and finally to invasive GC. A subsequent study by Correa evaluated the gastric precancerous process in a Colombian population (7). The results of this cross-sectional study led to the widely accepted conclusion that the severity of atrophy correlates with the prevalence of metaplasia and that the severity of metaplasia correlates with the prevalence of dysplasia, suggesting that the process is indeed a biological continuum (69).

Given that inflammation is a hallmark of gastric carcinogenesis, polymorphisms in genes encoding pro-inflammatory cytokines/chemokines have been the focus of much research in recent years. To date, polymorphisms in the interleukin (IL)-1 family genes have been the most widely studied, including polymorphisms in *IL1A, IL1B*, and *IL1RN* that encode IL-1α, IL-1β, and their endogenous receptor antagonist IL-1RA, respectively. In particular, IL-1β, a potent endogenous pyrogen and an important component in the development of Th2-mediated immunity (70, 71), has been associated with lipid peroxidation, DNA damage, inhibition of gastric acid secretion, increased *H. pylori* colonization, and induction of gastric atrophy and dysplasia in the presence or absence of *H. pylori* (72). Global meta-analyses have shown that the *IL1B-511* T allele is significantly associated with an increased risk of developing GC in Caucasians but not Asians or Mestizos (73, 74). Furthermore, IL-1 receptor signaling is known to induce the production of genes that not only stimulate tumor growth but are also involved in angiogenesis and metastasis such as matrix metalloproteinases, basic fibroblast growth factor, vascular endothelial growth factor, vascular cell adhesion molecule 1, intercellular adhesion molecule 1, monocytic chemotactic protein 1, and CXCL-2 (75). To date, only one study has addressed the role of *IL1R1* (also known as *CD121A*) in GC and *H. pylori* infection. The study, conducted in a Caucasian population, showed an increased risk of *H. pylori* infection in those harboring the *IL1R1* Hinfl A allele (OR: 2.01, *P*-value: 0.009) but failed to show an association with GC (76). In addition, a recent meta-analysis on the endogenous receptor antagonist IL-1RA has shown the IL1RN*22 genotype to increase the risk of gastric precancerous lesions, supporting a role for this polymorphism in the early stages of gastric carcinogenesis (OR: 2.27, 95% CI: 1.40–3.70) (77). A further meta-analysis that included 39 case–control studies, showed statistically significant associations between the IL1RN*22 genotype and both intestinal-type and diffuse-type GC, showing ORs of 1.83 and 1.72, respectively (78). Further examples of polymorphisms in pro-inflammatory cytokines/chemokines that play an essential role promoting inflammation in the context of gastrointestinal carcinogenesis are IL-4 (*IL4*-590C/T and -168T/C) (79), IL-6 (*IL6*-174 G/C) (80–82), IL-8 (*IL8*-251 A/T, +396 T/G, and +781 C/T) (79, 83), IL-10 (*IL10*-1082 A/G, −819 C/T, and −592 C/A) (84–86), IL-12 (*IL12A*-701 C/A, −798 T/A, +277 G/A, and −504 T/G) (87), IL-17 (*IL17*-197 G/A and +7488 T/C) (79), IL-18 (*IL18*-137 G/C) (88), and TNF-α (*TNFA* −238 G/A, −308 G/A, and −857 C/T) (89). In addition to this, a recent comprehensive review on this topic recommended the investigation of other polymorphisms in *IL1B* (3954 C/T and −1473G/C), *IL4* (–168T/C), *IL6* (572 G/C and 597 G/A), and *IL17* (+7488A/G and −197G/A), given their potential relevance in GC (79).

While extensive evidence supports the important role of pro-inflammatory cytokines/chemokines in gastric carcinogenesis, given that PRRs, mainly TLRs and NLRs, are important modulators of intestinal epithelial barrier function, epithelial repair, and immune homeostasis in the gastrointestinal tract (90), and that signal transduction from these receptors converges upon a common set of signaling molecules, including the activation of the transcription factors nuclear factor kappa-light-chain-enhancer of activated B cells (NF-κB) and the activator protein 1 (AP-1) that lead to the production of pro-inflammatory cytokines/chemokines (e.g., IL-1α, IL-1β, IL-6, IL-8, IL-10, and TNF-α) as well as members of the interferon (IFN) regulatory transcription factor family that mediate type I IFN-dependent responses, defects in PRRs function could be even more important than defects in pro-inflammatory cytokines/chemokines *per se* in the instauration of an inflammation-related disorder such as GC.

## Pattern-Recognition Receptors in Gastric Cancer

Innate immunity refers to responses that do not require previous exposure to an immune stimulus and represents the first line of host defense in the response to pathogens. PRRs are part of the innate immune system and are pivotal for the detection of invariant microbial motifs. PRRs have been divided into five distinct genetic and functional clades: TLRs, NLRs, C-type lectin receptors (CLRs), retinoic acid-inducible gene (RIG)-I-like receptors (RLRs), and absent in melanoma 2 (AIM2)-like receptors (ALRs) (91, 92). PRRs are commonly expressed by cells of the innate immune system such as monocytes, macrophages, dendritic cells (DCs), neutrophils, and epithelial cells, as well as cells of the adaptive immune system (93).

Toll-like receptors and CLRs scan the extracellular milieu and endosomal compartments for pathogen-associated molecular patterns (PAMPs), which are highly conserved microbial structures that are essential for microbial survival (94), while intracellular PRRs, including NLRs, RLRs, and ALRs, cooperate to provide cytosolic surveillance (92, 93).

**Figure 2:**
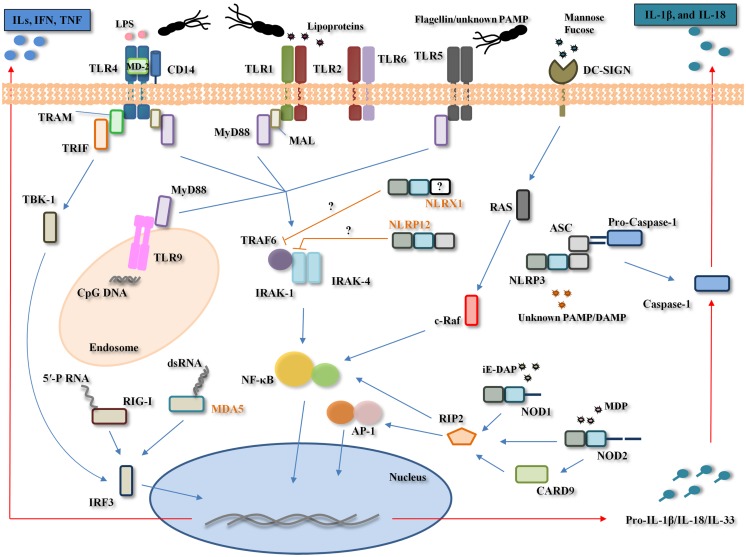
**Pattern-recognition receptors involvement in *Helicobacter pylori* infection**. *H. pylori* is recognized by the Toll-like receptors (TLRs) (TLR2, TLR4, TLR5, and TLR9), NOD-like receptors (NLRs) (NOD1, NOD2, NLRP3, and possibly, NLRP12 and NLRX1), RIG-I like receptors (RLRs) (RIG-I and possibly, MDA-5), and C-type lectin receptors (CLRs) (DC-SIGN). TLR4 poorly recognizes *H. pylori* lipopolysaccharide (LPS) to generate pro-inflammatory cytokines (e.g., IL-1α, IL-1β, IL-6, IL-8, IL-10, and TNF-α) and interferons (IFNs) through the myeloid differentiation primary response gene 88 (MyD88)-dependent and -independent pathways, respectively. TLR2 recognizes *H. pylori* LPS/peptigoglycan/unknown pathogen-associated molecular pattern (PAMP) while TLR5 poorly recognizes *H. pylori* flagella and TLR9 recognizes *H. pylori* DNA (unmethylated CpG motifs). *H. pylori* recognition by these three TLRs leads to nuclear factor-κB (NF-κB) activation. NOD1 and NOD2 recognize *H. pylori* peptidoglycan-derived peptides [γ-d-glutamyl-meso-diaminopimelic acid (iE-DAP) and muramyl dipeptide (MDP)], leading to the activation of both transcription factors NF-κB and activator protein (AP)-1. The NLRP3 inflammasome, comprising NLRP3, apoptosis-associated speck-like protein containing a CARD (ASC) and caspase-1, recognizes a yet unknown *H. pylori* PAMP and/or damage-associated molecular pattern (DAMP), and through caspase-1 cleavage, leads to the maturation and secretion of interleukin (IL)-1β and IL-18. NLRX1 and NLRP12, two known negative regulators of NF-κB, appear to be significantly down-regulated during *H. pylori* infection *in vitro*, however, their exact role during *H. pylori* infection remains unclear. RIG-I recognizes *H. pylori* 5′-triphosphorylated RNA (5′-PRNA) while MDA-5 possibly recognizes *H. pylori* dsRNA. The dendritic cell-specific intercellular adhesion molecule-3 grabbing non-integrin (DC-SIGN) recognizes *H. pylori* fucosylated ligands and this interaction appears to counteract the pro-inflammatory immune response to *H. pylori*. Only one generic cell type depicting all TLRs, NLRs, RLRs, and CLRs involved in *H. pylori* recognition is shown here for simplicity. MAL, MyD88 adaptor-like protein, also named TIRAP; TRAM, translocating chain-associating membrane protein; TRIF, TIR domain containing adaptor inducing interferon-beta protein; TBK-1, TANK-binding kinase 1; IRF3, IFN-regulatory factor 3; TRAF6, TNF receptor-associated factor 6; IRAK, interleukin 1 receptor-associated kinase; RAS, proto-oncogene ras; c-RAF, proto-oncogene protein ras; RIP2, receptor-interacting serine/threonine-protein kinase 2, also known as RICK; CARD9, caspase activation and recruitment domain; MD-2, myeloid differentiation protein-2; ILs: interleukins. Names in orange correspond to molecules with a probable but not established role in the host response to *H. pylori*.

In *H. pylori* infection, the first physical–chemical barriers for the pathogen are the mucus layer, gastric epithelial cells, autophagy, and PRRs (TLRs, NLRs, CLRs, and RLRs) (Figure 2).

## Toll-Like Receptors and *Helicobacter pylori*-Related Gastric Cancer

### Toll-like receptors recognition of *Helicobacter pylori*

The involvement of the TLR signaling pathway in infectious, autoimmune, and inflammatory diseases is well accepted (95). During *H. pylori* infection, TLRs on gastric epithelial and immune cells recognize diverse PAMPs such as flagellin/unknown PAMP (TLR5), unmethylated CpG motifs (TLR9), and lipopolysaccharide (LPS) (TLR4 and TLR2).

TLR4 was initially identified as the potential signaling receptor for *H. pylori* LPS on gastric epithelial cells (96–99). After forming a complex with the LPS-binding protein (LBP), LPS interacts with the monocyte differentiation antigen CD14 (CD14), and subsequently with the myeloid differentiation protein-2 (MD-2) (100). Together with TLR4, this complex induces the TLR4-mediated MyD88-dependent signal transduction pathway, which leads to the rapid activation of transcription factors, mainly NF-κB, and cytokines such as TNF-α, IL-1β, IL-6, and IL-12 (95). On the other hand, stimulation of TLR4 by LPS also facilitates the activation of a MyD88-independent pathway that activates IFN-regulatory factor (IRF) 3 and involves the late phase of NF-κB activation, both of which lead to the production of IFN-β and the expression of IFN-inducible genes (101, 102). In addition to LPS, the *H. pylori* secretory protein HP0175, through its ability to bind to TLR4, was shown to transactivate the epidermal growth factor receptor (EGFR) and stimulate the EGFR-dependent vascular endothelial growth factor production in the GC cell line AGS, which have been linked to *H. pylori*-associated gastroduodenal diseases, ulcerogenesis, and carcinogenesis (103).

Although early studies concluded that TLR4 is the first innate immune response against *H. pylori* (104, 105), later studies suggested that TLR4 had a limited role, given that *H. pylori* LPS appeared to bind poorly to LBP, resulting in it being inefficiently transferred to CD14 (106). Consequently, recent studies addressing the role of other TLRs during *H. pylori* infection, have found TLR2 to be the initial barrier against *H. pylori* infection (107–112). A potential explanation for these inter-study differences in relation to the TLRs response to *H. pylori* might be attributed to cell type (i.e., epithelial versus immune cells), origin of the cell studied (i.e., peritoneal versus bone marrow derived macrophages), and the type of inflammatory response measured (i.e., type of cytokines), and thus, currently any conclusions regarding the role of TLR4 must be treated with caution.

In contrast, there is strong evidence supporting an important role for TLR2 in *H. pylori* infection, with both animal and cell culture experiments suggesting that TLR2 ligands (LPS or other) exist in *H. pylori* and related *Helicobacter* species (112–114), and that TLR2 may be involved in the innate immune sensing of these bacteria by epithelial cells (113). Furthermore, an interesting publication by Smith et al. (115) showed that *H. pylori* LPS functions as a classic TLR2 ligand and induces a discrete pattern of chemokine expression in epithelial cells, which involves modulation of the expression of the signaling protein tribbles 3 (TRIB3), a molecule implicated in the regulation of NF-κB.

Yet, the most likely scenario is that both TLR4 and TLR2 are involved in the early immune response against *H. pylori* as has been demonstrated by a number of investigators (116–118). For example, Obonyo et al. (116) showed that both TLR2 and TLR4 were crucial signaling receptors for *H. pylori* activation of the host immune response leading to the secretion of cytokines. Further, Yokota et al. (118) not only showed that *H. pylori* LPS was initially targeted by TLR2 as described by others, but, for the first time, showed that this TLR2 activation leads to cell proliferation and TLR4 expression via the MEK1/2-ERK1/2 pathway. The final outcome of this signaling pathway is increased proliferation of gastric epithelial cells and the instauration of a strong inflammatory reaction. Once this response is instaurated, *H. pylori* could then enhance inflammatory reactions mediated by TLR4 agonists such as other bacterial LPS, which would also contribute to gastric inflammation and subsequent carcinogenesis (118). Further, the heat-shock protein 60, an immune-potent antigen of *H. pylori*, has been shown to activate NF-κB and induce IL-8 production through TLR2 and TLR4 pathways in gastric epithelial cells, a phenomenon that is likely to contribute to the development of gastric inflammation caused by *H. pylori* infection (117).

In addition, TLR9 appears to play an important role in *H. pylori* recognition. Interestingly, Rad et al. (112) identified TLR9-mediated recognition of *H. pylori* DNA as a main *H. pylori*-induced intracellular TLR signaling pathway in DCs. Further, a study using a murine model of *H. pylori* infection has suggested that TLR9 signaling is involved in the suppression of *H. pylori*-induced gastritis in the early phase of infection via down-regulation of Th1-type cytokines modulated by IFN-α (119). In addition, a recent study has shown that the gastric epithelia of children respond to *H. pylori* infection by increasing the expression of TLR2, TLR4, TLR5, and TLR9, as well as the cytokines IL-8, IL-10, and TNF-α (120).

Although TLR5 interaction with *H. pylori* induces only weak receptor activation (121), TLR5 has been involved in the inflammatory response to *H. pylori*. An interesting publication by Smith et al. (107), using HEK293 cells transfected with specific TLR expression constructs and MKN45 cells expressing dominant negative versions of TLR2, TLR4, and TLR5, which block the activity of wild-type forms of these receptors, has demonstrated that live *H. pylori* induces NF-κB activation and chemokine gene expression due to ligation of TLR2 and TLR5. A further study that aimed to explore the involvement of TLR2 and TLR5 in THP-1 cells and HEK293 cell lines (stably transfected with TLR2 or TLR5) during *H. pylori* infection, has indicated that *H. pylori*-induced expression of TLR2 and TLR5 can qualitatively shift *cag* PAI-dependent to *cag* PAI-independent pro-inflammatory signaling pathways with possible impact on the outcome of *H. pylori*-associated diseases (122). Given the established TLR5 evasion of α and ε *Proteobacteria* including *H. pylori* (123), the TLR5-mediated inflammatory responses during *H. pylori* infection described by Smith et al. (107) and Kumar Pachathundikandi et al. (122) are likely to be flagellin-independent, and therefore, a still unknown *H. pylori* factor might be responsible for this.

The importance of TLRs recognition during *H. pylori* infection and GC development is further supported by the acquired characteristics that enable *H. pylori* to survive in the human stomach and cause chronic inflammation. For example, *H. pylori* LPS is characterized by a modification of the lipid A component of LPS that makes it less pro-inflammatory (124) and has been reported to exhibit a 1000-fold reduction in bioactivity as compared to *Escherichia coli* LPS (125). Also, the flagellin of this bacterium has been shown to be poorly recognized due to modifications in the TLR5 recognition site of the N-terminal D1 domain of flagellin (123).

### Toll-like receptors and gastric carcinogenesis

While TLR2, TLR4, TLR5, and TLR9 appear to be important for *H. pylori* recognition, their role in the evolution of gastritis to more advanced lesions remains unclear. Interestingly, Schmausser et al. (126) showed that TLR9 was not detectable in intestinal metaplasia or dysplasia and was only focally detected in 6 out of 22 gastric carcinomas, while TLR4 and TLR5 were strongly expressed by gastric carcinomas. Consistently, a study by Pimentel-Nunes et al. (127) showed a statistically significant trend for a progressive increase of TLR2, TLR4, and TLR5 expression from normal mucosa to gastric dysplasia (mean expression in normal mucosa: 0.1, gastritis: 1.0, metaplasia: 2.2, and dysplasia: 2.8, *P*-value <0.01), with dysplasia presenting more than 90% positive epithelial cells showing strong expression (2.8, 95% CI: 2.7–3). In addition, these authors showed a significant trend for decrease in TOLLIP and PPARγ, two TLR signaling pathway inhibitors, which was associated with increasing levels of CDX-2, a marker for adenocarcinoma, from normal mucosa to carcinoma (*P*-value <0.05) (128). Fernandez-Garcia et al. (129) have also reported increased expression of TLR3, TLR4, and TLR9 in GC, and furthermore, these authors noted that TLR3 expression by cancer cells was significantly associated with a poor overall survival in patients with resectable tumors, which lead them to suggest that TLR3 might be an indicator of tumor aggressiveness. Similarly, Yakut et al. (130) investigating the association between serum IL-1β, TLR4 levels, pepsinogen I and II, gastrin 17, vascular endothelial growth factor, and *H. pylori* CagA status in patients with a range of gastric precancerous lesions, concluded that serum TLR4 levels could be used as a biomarker to differentiate individuals presenting with dysplasia from those with other gastric precancerous lesions, the mean TLR4 level in patients with dysplasia (0.56 ± 0.098 ng/mL) being significantly higher than in patients with *H. pylori* positive chronic non-atrophic gastritis (0.10 ± 0.15 ng/mL), chronic atrophic gastritis (0.06 ± 0.07 ng/mL), and intestinal metaplasia (0.12 ± 0.18 ng/mL). Furthermore, while TLRs have been shown to be expressed at the apical and basolateral pole of both normal gastric epithelial cells and in *H. pylori* gastritis, in metaplasia, dysplastic, and neoplastic epithelial cells all TLRs are expressed diffusely and homogeneously throughout the cytoplasm, with no apparent polarization, which may suggest an increased activation of these diffusely over-expressed receptors during gastric carcinogenesis (126, 128).

In recent years, TLRs have been associated with tumor development and progression processes including cell proliferation, epithelial–mesenchymal transition, angiogenesis, metastasis, and immunosuppression. Interestingly, Chochi et al. (104) not only showed that *H. pylori* augmented the growth of GC via the LPS-TLR4 pathway but also found that this bacterium attenuated the antitumor activity and IFN-γ-mediated cellular immunity of human mononuclear cells. In addition, Song et al. (131) have suggested that flagellin-activated TLR5 enhances the proliferation of GC cells through an ERK-dependent pathway. Furthermore, Tye et al. (132) have proposed a novel role for TLR2 in promoting gastric tumorigenesis independent of inflammation, whereby up-regulation of TLR2 within epithelial tumor cells, rather than infiltrating inflammatory cells, by the uncontrolled activation of the oncogenic transcription factor STAT3, promoted gastric tumor cell proliferation, and survival via up-regulation of anti-apoptotic genes [e.g., BCL2-related protein A1 (*BCL2A1*), baculoviral IAP repeat containing 3 (*BIRC3*), and B-cell CLL/lymphoma 3 (*BCL3*)]. Further, two processes that facilitate carcinogenesis and involve TLRs have recently been described by Li et al. (133). Using LPS-treated CD14-knockdown GC cells, these authors showed that CD14, an important co-receptor in the TLR4 complex, promotes tumor cell epithelial–mesenchymal transition and invasion through TNF-α (133).

In addition, the expression of tumor-associated molecules known to be important in gastric carcinogenesis has been linked to the activation of the TLR signaling pathway. For example, prostaglandin-endoperoxide synthase 2 (PTGS2), which is also termed cyclooxygenase 2 (COX2), a key enzyme that catalyzes the conversion of arachidonic acid to prostaglandins, has been shown to play a pivotal role in gastric inflammation and carcinogenesis (134). For example, a study by Chang et al. (108), using clinical *H. pylori* isolates, has shown that *H. pylori* acts through TLR2/TLR9 to activate both the PI-PLCγ/PKCα/c-Src/IKKα/β and NIK/IKKα/β pathways, resulting in the phosphorylation and degradation of IκBα, which in turn leads to the stimulation of NF-κB and the expression of PTGS2.

Further, as compared with normal cells, cancer cells are more metabolically active and generate more reactive oxygen species (ROS), which affects cell survival. Several studies have suggested that ROS can act as secondary messengers and control a range of signaling cascades, leading to sustained proliferation of cancer cells (135, 136). In the context of gastric carcinogenesis, *H. pylori*-infected gastric epithelial cells have been shown to generate ROS (137). Interestingly, Yuan et al. (138) recently suggested that TLR4 expression in GC correlated with tumor stage and that activation of TLR4 contributed to GC cell proliferation via mitochondrial ROS production through up-regulation of phosphorylated Akt and NF-κB p65 activation and nuclear translocation.

However, the involvement of TLRs in GC might be more complex than initially suspected as TLRs not only recognize antigenic determinants of viruses, bacteria, protozoa, and fungi, but are also involved in the detection of damage-associated molecular patterns (DAMPs) (e.g., extracellular adenosine triphosphate, hyaluran, extracellular glucose, monosodium urate crystals) (139). Release of DAMPs, which are especially targeted by TLR2 and TLR4 (140–145) during cancer progression may cause chronic inflammation leading to down-regulation of the ζ chain of the T-cell and NK cell activating receptors [for comprehensive information on this topic see the review by Baniyash et al. (146)], which entails T-cell and NK cell dysfunction, a phenomenon observed in some malignancies such as GC (147, 148), colon (149), prostate (150), cervical (151), and pancreatic cancer (152). In addition to immunosuppression, DAMPS appear to facilitate other processes during gastric carcinogenesis. For example, Wu et al. (153) have recently showed that hyaluronan, derived from malignant cells, induced long-lived tumor-associated neutrophils and subsequent malignant cell migration in gastric carcinomas via a TLR4/PI3K interaction.

Collectively, TLRs might be involved in both gastric carcinogenesis mediated by *H. pylori* infection (a tumor-promoting consequence of inflammatory responses) and in GC perpetuation associated with immunosuppression (active evasion by cancer cells from attack and elimination by immune cells) and increased metastasis.

### Genetic polymorphisms involved in the toll-like receptor signaling pathway and gastric cancer

In recent years, a number of investigations have attempted to establish the relationship between polymorphisms in molecules of the TLR signaling pathway and risk of GC. Recent studies, conducted in several populations, have shown associations between the polymorphisms *TLR1* rs5743618 (Ile602Ser) (154), *TLR2* −196 to −174del (155–158), *TLR2* rs3804099 (157), *TLR4* rs4986790 (Asp299Gly) (155, 157, 159), *TLR4* rs4986791 (Thr399Ile) (160), *TLR4* rs10116253 (161), *TLR4* rs10983755 (162), *TLR4* rs11536889 (+3725G/C) (155), *TLR4* rs1927911 (161), *TLR5* rs5744174 (158), *TLR9* rs187084 (−1486 T/C) (163), and *CD14* rs2569190 (−260 C/T) (155, 164–167), and risk of GC development in an ethnic-specific manner (Table 1). In addition, three polymorphisms located in the *TLR4* mRNA promoter region (sites −2081, −2026, and −1601) and *TLR4* Thr135Ala at the leucine-rich repeat (LRR), have been associated with poorly differentiated GC (168, 169).

**Table 1:** **Genetic polymorphisms in the Toll-like receptor signalling pathway that have been studied in relation to gastric cancer (170)**.

Gene	Polymorphism	Reference	Population	GC subtype	Total sample size	OR, 95% CI^a^
*TLR1*	rs5743618 (Ile602Ser)	Yang et al. (154)	German	NS	284^b^	OR: 0.40, 95% CI: 0.22–0.72
*TLR2*	−196 to −174del	Castaño-Rodríguez et al. (170)	Chinese	Non-cardia	310	OR: 1.17, 95% CI: 0.81–1.71
		de Oliveira et al. (157)	Brazilian	Non-cardia	440	OR: 2.32, 95% CI: 1.56–3.46
		Zeng et al. (158)	Chinese	NS	744	OR: 0.66, 95% CI: 0.48–0.90
		Hishida et al. (172)	Japanese	NS	1680	OR: 1.17, 95% CI: 0.79–1.73^c^
		Tahara et al. (156)	Japanese	Non-cardia	744	OR: 6.06, 95% CI: 1.86–19.72
	rs3804099	de Oliveira et al. (157)	Brazilian	Non-cardia	440	OR: 2.32, 95% CI: 1.56–3.46
	rs3804100	Castaño-Rodríguez et al. (170)	Chinese	Non-cardia	310	OR: 3.16, 95% CI: 1.38–7.24
*TLR4*	rs4986790 (Asp299Gly)	Qadri et al. (174)	Indian	NS	330	OR: 1.15, 95% CI: 0.66–2.03
		de Oliveira et al. (157)	Brazilian	Non-cardia	440	OR: 2.01, 95% CI: 1.06–3.81
		Schmidt et al. (175)	Chinese	Non-cardia	222	OR: 0.23, 95% CI: 0.03–1.81
		Santini et al. (160)	Italian	NS	322	OR: 0.97, 95% CI: 0.37–1.14
		Trejo de la O (176)	Mexican	NS	182	OR: 2.70, 95% CI: 0.36–10.70
		Hold et al. (159)	Caucasian^d^	Non-cardia	731	OR: 2.50, 95% CI: 1.60–4.00
		Hold et al. (159)	Caucasian^e^	Cardia and non-cardia	395	OR: 2.10, 95% CI: 1.10–4.20
		Garza-Gonzalez et al. (177)	Mexican	Non-cardia	314	OR: 1.00, 95% CI: 0.30–2.80
	rs4986791 (Thr399Ile)	Qadri et al. (174)	Indian	NS	330	OR: 1.39, 95% CI: 0.70–2.78
		de Oliveira et al. (157)	Brazilian	Non-cardia	440	OR: 1.81, 95% CI: 0.64–5.15
		Santini et al. (160)	Italian	NS	322	OR: 3.62, 95% CI: 1.27–6.01
		Trejo de la O (176)	Mexican	NS	263	OR: 1.40, 95% CI: 0.36–5.38
		Garza-Gonzalez et al. (177)	Mexican	Non-cardia	314	OR: 0.25, 95% CI: 0.01–1.80
	rs10116253	Castaño-Rodríguez et al. (170)	Chinese	Non-cardia	310	OR: 0.58, 95% CI: 0.34–1.00
		Huang et al. (161)	Chinese	NS	511	OR: 0.33, 95% CI: 0.18–0.60
	rs10759931	Castaño-Rodríguez et al. (170)	Chinese	Non-cardia	310	OR: 0.56, 95% CI: 0.33–0.97
	rs10759932	Castaño-Rodríguez et al. (170)	Chinese	Non-cardia	310	OR: 0.59, 95% CI: 0.34–1.04
		Huang et al. (178)	Chinese	Cardia and non-cardia	1962	OR: 1.03, 95% CI: 0.74–1.45
	rs10983755	Kim et al. (179)	Korean	Non-cardia	974	OR: 1.41, 95% CI: 1.01–1.97
	rs11536889	Castaño-Rodríguez et al. (170)	Chinese	Non-cardia	310	OR: 3.58, 95% CI: 1.20–10.65
		Kupcinskas et al. (180)	Caucasian^f^	NS	349	OR: 1.03, 95% CI: 0.62–1.71
		Hishida et al. (181)	Japanese	NS	1639	OR: 1.04, 95% CI: 0.66–1.63
	rs1927911	Castaño-Rodríguez et al. (170)	Chinese	Non-cardia	310	OR: 0.47, 95% CI: 0.27–0.82
		Huang et al. (161)	Chinese	NS	511	OR: 0.37, 95% CI: 0.21–0.70
	rs2149356	Castaño-Rodríguez et al. (170)	Chinese	Non-cardia	310	OR: 0.59, 95% CI: 0.34–1.02
*TLR5*	rs5744174	Zeng et al. (158)	Chinese	NS	744	OR: 1.43, 95% CI: 1.03–1.97
*TLR9*	rs187084 (−1486 T/C)	Wang et al. (163)	Chinese	Cardia and non-cardia	628	OR: 1.63, 95% CI: 1.01–2.64
*CD14*	rs2569190 (−260 C/T)	Castaño-Rodríguez et al. (170)	Chinese	Non-cardia	310	OR: 0.72, 95% CI: 0.5–1.02
		Companioni et al. (164)	Caucasian^g^	Cardia and non-cardia	1649	OR: 0.92, 95% CI: 0.77–1.09
		Li et al. (133)	Tibetan	NS	462	OR: 2.16, 95% CI: 1.34–3.47
		Kim et al. (179)	Korean	Non-cardia	974	OR: 0.97, 95% CI: 0.77–1.23^h^
		Hold et al. (182)	Caucasian^d^	Non-cardia	716	OR: 1.00, 95% CI: 0.70–1.40
		Hold et al. (182)	Caucasian^e^	Cardia and non-cardia	395	OR: 0.80, 95% CI: 0.50–1.30
		Tahara et al. (166)	Japanese	Non-cardia	237	OR: 0.31, 95% CI: 0.12–0.78
		Zhao et al. (167)	Chinese	NS	940	OR: 1.95, 95% CI: 1.20–3.16
		Wu et al. (183)	Chinese	Non-cardia	414	OR: 0.98, 95% CI: 0.75–1.29
*MD-2*	rs11465996	Castaño-Rodríguez et al. (170)	Chinese	Non-cardia	310	OR: 4.83, 95% CI: 2.02–11.57
	rs16938755	Castaño-Rodríguez et al. (170)	Chinese	Non-cardia	310	OR: 3.80, 95% CI: 1.48–9.77
*LBP*	rs2232578	Castaño-Rodríguez et al. (170)	Chinese	Non-cardia	310	OR: 3.07, 95% CI: 1.24–7.59
*TIRAP*	rs7932766	Castaño-Rodríguez et al. (170)	Chinese	Non-cardia	310	OR: 6.04, 95% CI: 1.89–19.36

*GC, gastric cancer; OR, odds ratio; CI, confidence intervals; NS, not specified*.

*^a^OR and 95% CI correspond to allele or genotype analysis, depending on available information in the article*.

*^b^The control group included individuals with high risk gastritis (pangastritis, corpus-predominant gastritis with or without the presence of gastric atrophy, and intestinal metaplasia in either antrum or corpus)*.

*^c^Compared to gastric atrophy controls*.

*^d^The study population is from Poland*.

*^e^The study population is from the United States. No significant association was found with cardia GC*.

*^f^Subjects from Germany, Lithuania and Latvia*.

*^g^Subjects from France, Italy, Spain, United Kingdom, The Netherlands, Greece, Germany, Sweden, Denmark and Norway*.

*^h^Effect size for intestinal-type GC, diffuse type: OR: 0.99, 95% CI: 0.78–1.26*.

Interestingly, some of these polymorphisms including *TLR4* Asp299Gly (159, 184), *TLR4* Thr399Ile (184, 185), *TLR4* rs10759932 (186), *CD14*-260 C/T (187), and *TLR2* −196 to −174del (157), appear to be involved in the biological continuum that results in intestinal-type GC as they have also been associated with gastric precancerous lesions (Table 2).

**Table 2:** **Genetic polymorphisms in the Toll-like receptor signalling pathway that have been studied in relation to gastric precancerous lesions**.

Reference	Journal	Population	Precancerous lesion	Cases	Controls	Total	Polymorphism	OR (95% CI)^a^
Fan et al. (186)	Human Immunology	Chinese	IM	193	312	505	*TLR4* Asp299Gly	0.89 (0.46–1.72)
							*TLR4* Thr399Ile	1.01 (0.33–3.14)
							*TLR4* rs10759932	0.42 (0.29–0.62)
			Dysplasia	140	312	452	*TLR4* Asp299Gly	0.81 (0.38–1.73)
							*TLR4* Thr399Ile	0.83 (0.22–3.19)
							*TLR4* rs10759932	0.62 (0.41–0.93)
de Oliveira et al. (157)	Digestive Diseases and Science	Brazilian	CG	229	240	469	*TLR4* Asp299Gly	1.60 (0.84–3.06)
							*TLR4* Thr399Ile	1.08 (0.35–3.39)
							*TLR2* −196–174 del	1.52 (1.01–2.29)
Kupcinskas et al. (180)	BMC Medical Genetics	Caucasian	CG, AG and IM	222	238	460	*TLR4* rs11536889	0.94 (0.62–1.44)
Zeng et al. (158)	Cancer Epidemiology, Biomarkers and Prevention	Chinese	IM	496	496	992	*TLR2* −196–174 del	0.99 (0.65–1.52)
							*TLR5* rs5744174	1.55 (0.78–3.11)
			Dysplasia	350	496	846	*TLR2* −196–174 del	0.99 (0.73–1.35)
							*TLR5* rs5744174	1.73 (0.84–3.55)
Rigoli et al. (184)	Anti-Cancer Research	Caucasian	CG	60^b^	87	147	*TLR4* Asp299Gly	4.80 (1.93–12.35)
							*TLR4* Thr399Ile	3.73 (1.36–10.14)
Hishida et al. (172)	Gastric Cancer	Japanese	AG^c^	494	443	937	*TLR2* −196–174 del	1.08 (0.70–1.67)
Hishida et al. (181)	Helicobacter	Japanese	AG^c^	536	1056	1592	*TLR4* rs11536889	1.20 (0.76–1.89)
Murphy et al. (188)	European Journal of Gastroenterology and Hepatology	Caucasian	CG	91	96	187	*TLR4* Asp299Gly	1.12 (0.49–2.52)
				90	91	181	*TLR4* Thr399Ile	0.97 (0.44–2.11)
			IM	63	96	159	*TLR4* Asp299Gly	1.33 (0.49–3.59)
				62	91	153	*TLR4* Thr399Ile	0.99 (0.38–2.63)
Hofner et al. (189)	Helicobacter	Caucasian	CG	136^d^	75	211	*TLR4* Asp299Gly	1.25 (0.53–2.95)
							*TLR4* Thr399Ile	0.94 (0.39–2.24)
Achyut et al. (185)	Human Immunology	Indian	AG	68	200	268	*TLR4* Asp299Gly	1.50 (0.55–3.82)
							*TLR4* Thr399Ile	4.2 (1.13–15.73)
			IM	50	200	250	*TLR4* Asp299Gly	1.10 (0.32–3.50)
							*TLR4* Thr399Ile	4.7 (1.52–14.63)
Hold et al. (159)	Gastroenterology	Caucasian	AG	45^e^	100	145	*TLR4* Asp299Gly	11.0 (2.50–48.0)
Kato et al. (187)	Digestive Diseases and Science	Venezuelan	AG	289	1033	1322	*CD14* −260 C/T	1.17 (0.81–1.70)
			IM	543	1033	1575	*CD14* −260 C/T	1.45 (1.06–1.99)
			Dysplasia	118	1033	1151	*CD14* −260 C/T	1.44 (0.82–2.55)

*CG, chronic gastritis; AG, atrophic gastritis; IM, intestinal metaplasia; OR, odds ratio; CI, confidence intervals*.

*^a^OR and 95% CI correspond to allele or genotype analysis, depending on available information in the article*.

*^b^Only individuals with corpus-predominant chronic gastritis were included in the meta-analysis (individual presenting antrum-predominant gastritis were excluded)*.

*^c^Analyses including only *H. pylori* seropositive individuals*.

*^d^Only patients with chronic gastritis were included in the meta-analysis (patients presenting duodenal ulcer were excluded)*.

*^e^Cases were GC patients’ relatives with gastric atrophy and infected with *H. pylori* from a Scotland population*.

Given that some authors have failed to show specific associations between polymorphisms in the TLR signaling pathway, especially in *TLR2, TLR4*, and *CD14*, and gastric precancerous lesions/GC (157, 160, 162, 164, 172, 174–178, 180–183, 185, 188, 189), we performed the first global meta-analysis to assess the role of *TLR2, TLR4*, and *CD14* polymorphisms in gastric carcinogenesis (155), in an attempt to clarify the limited and current conflicting evidence, and to establish the true impact of the TLR signaling pathway in GC. Our meta-analysis, which included 18 case–control studies conducted in Caucasian, Asian, and Latin American populations, showed that *TLR4* Asp299Gly was a definitive risk factor for GC in Western populations (pooled OR: 1.87, 95% CI: 1.31–2.65). In addition, there was a potential association between *TLR2* −196 to −174 and GC in Japanese (pooled OR: 1.18, 95% CI: 0.96–1.45) (155). Interestingly, a recent meta-analysis on *TLR2* −196 to −174 and the risk of GC, conducted by Cheng et al. (190), failed to reproduce the findings in our meta-analysis, however, their stratification by ethnicity analyses included subjects from both Japan and China, which might explain the different outcomes. A further meta-analysis conducted by Chen et al. (191) that included 21 case–control studies showed an overall increased risk of GC in individuals harboring *TLR4* Asp299Gly (Allele analysis, OR: 1.84, 95% CI: 1.41–2.39) and *TLR4* Thr399Ile (Allele analysis, OR: 1.97, 95% CI: 1.22–3.18). Consistently, in stratified analyses by ethnicity, these authors only found an association between *TLR4* Asp299Gly (Allele analysis, OR: 1.90, 95% CI: 1.43–2.51) and *TLR4* Thr399Ile (Allele analysis, OR: 2.84, 95% CI: 1.56–5.15) in Caucasian individuals (191). Further, Zhao et al. (192) in an updated version of a meta-analysis that was initially conducted by Zhang et al. (193), on the risk of *TLR4* polymorphisms and risk of cancer in general, found a significant association with GC after stratifying by cancer type (OR: 2.00, 95% CI: 1.53–2.62). In addition, Zou et al. (194), through a meta-analysis that included 10 case–control studies, not only found that *TLR4* Asp299Gly was associated with GC (OR: 1.87, 95% CI: 1.44–2.44), especially non-cardia GC (OR: 2.03, 95% CI: 1.51–2.72), but also gastric precancerous lesions (OR: 2.47, 95% CI: 1.57–3.88), especially in *H. pylori*-infected individuals (OR: 3.43, 95% CI: 1.92–6.13).

Given limited evidence regarding the association between polymorphisms in other molecules of the TLR signaling pathway and the risk of GC, and the fact that 42% of cases of GC worldwide occur in the Chinese population, we conducted a case–control study comprising 310 ethnic Chinese individuals (87 non-cardia GC cases and 223 controls with functional dyspepsia), in which 25 polymorphisms involved in the TLR signaling pathway were investigated (170). Seven polymorphisms showed significant associations with GC (*TLR4* rs11536889, *TLR4* rs10759931, *TLR4* rs1927911, *TLR4* rs10116253, *TLR4* rs10759932, *TLR4* rs2149356, and *CD14* −260 C/T). In multivariate analyses, *TLR4* rs11536889 remained a risk factor for GC even after adjustment (OR: 3.58, 95% CI: 1.20–10.65). Further, *TLR4* rs10759932 decreased the risk of *H. pylori* infection (OR: 0.59, 95% CI: 0.41–0.86) (170). Strikingly, statistical analyses assessing the joint effect of *H. pylori* and the selected polymorphisms revealed that *H. pylori*-infected individuals harboring *TLR2* rs3804100, *TLR2* −196 to −174del, *TLR4* rs11536889, *MD-2* rs11465996, *MD-2* rs16938755, *LBP* rs2232578, and *TIRAP* rs7932766 were at most risk of developing GC (Table 1) (170).

The functional relevance of a number of these polymorphisms has already been established. For example, two polymorphisms in *TLR4*, Asp299Gly, and Thr399Ile, have been shown to disrupt the normal structure of the extracellular domain of TLR4, and thus, as a result, may reduce responsiveness to *H. pylori* by diminishing the binding affinity of the bacterial ligands (195). In addition, the *TLR4* rs11536889 polymorphism, which is located in the center of the 2818-bp *TLR4* 3′ untranslated region (UTR), has recently been shown by Sato et al. (196) to contribute to the translational regulation of *TLR4*, possibly by binding to microRNAs. Further, these authors elegantly demonstrated that subjects harboring *TLR4* rs11536889 exhibited higher levels of TLR4 receptors on monocytes and secreted higher levels of IL-8 in response to LPS (196). In addition, *TLR4* rs10759932 has been shown to decrease the expression of forkhead box protein P3 (FOXP3), the most specific marker for natural regulatory T (Treg) cells (197). FOXP3+ Treg cells, which suppress the immune response of antigen-specific T cells, have been demonstrated to play a key role in immunologic tolerance (198). Notably, recent studies have not only shown that *in vivo* depletion of FOXP3+ Treg cells in *H. pylori*-infected mice leads to increased gastric inflammation and reduced bacterial colonization (199), but also recruitment of FOXP3+ Treg cells is increased in *H. pylori*-related human disorders including gastritis (200, 201), duodenal ulcer (202), and GC (200, 203, 204), suggesting that FOXP3+ Treg cells might contribute to lifelong persistence of *H. pylori* infection. Also, *TLR1* rs5743618 appears to impair the surface expression of TLR1 of NK cells and NK cells-derived IFN-γ production (154). Further, *TLR2* −196 to −174 has been associated with decreased transcriptional activity of *TLR2* (205, 206). Similarly, it has been demonstrated that *TLR9* rs187084 down-regulates TLR9 expression (207).

Further, CD14 has been shown to activate macrophages/monocytes to release Th1-type cytokines including IL-12, thus, establishing the chronic inflammation stimulated by *H. pylori* infection (208–210). A Th1 predominant response has been extensively associated with the pathogenesis of *H. pylori*-related gastric disease (211–213). Currently, however, controversy exists regarding the influence of *CD14* −260 on expression of soluble CD14 (sCD14). According to a number of studies, the *CD14* −260 T allele is believed to increase sCD14 production and therefore, serum sCD14 levels (214–217). In contrast, it has been reported that elevated sCD14 levels are associated with *H. pylori* infection, especially in subjects with the *CD14* −260 CC genotype (167). Alternatively, others have argued that this polymorphism has no effect on transcription (218). Since the evidence to date is conflicting, more functional studies are required to clarify this issue.

Overall, it is clear that genetic variability in genes of the TLR signaling pathway plays an important role in GC pathogenesis. Investigations of polymorphisms in different molecules of this pathway among different populations could provide novel insights into targeted treatment in genetically susceptible individuals, and thus, improve primary and secondary prevention of *H. pylori*-related GC in high risk populations.

## NOD-Like Receptors and *Helicobacter pylori*-Related Gastric Cancer

### NOD-like receptors recognition of *Helicobacter pylori*

The NLR family not only recognizes PAMPs but also DAMPs in the cytoplasm (93). The NLRs characteristic structure includes a central nucleotide-binding and oligomerization (NACHT) domain that is present in all NLR family members, a C-terminal LRRs and an N-terminal caspase recruitment (CARD) or pyrin (PYD) domain.

Based on phylogenetic analysis of NACHT domains, the NLR family has been shown to comprise three subfamilies: (1) the NOD family which includes NOD1-2, NOD 3 (NLRC3), NOD4 (NLRC5), NOD5 (NLRX1), and CIITA, (2) the NLRPs including NLRP1-14 (also known as NALPs), and (3) the IPAF subfamily, which consists of IPAF (NLRC4) and NAIP (93).

The NACHT domain belongs to a family of P-loop NTPases known as the signal transduction ATPases with numerous domains (STAND) (219). This domain permits activation of the signaling complex via adenosine ATP-dependent oligomerization (94). NACHT domain oligomerization is essential for the activation of NLRs, forming high molecular weight complexes, probably hexamers or heptamers that characterize inflammasomes (molecular complexes involved in the activation of inflammatory caspases for the maturation and secretion of IL-1β, IL-18, and possibly IL-33) and NOD signalosomes (complexes that are assembled upon oligomerization of NOD1 or NOD2 and lead to NF-κB activation through the receptor-interacting protein-2) (94). CARD and PYD are death domains that mediate homotypic protein–protein interactions for down-stream signaling (93, 94). These domains are characterized by six α helices that form trimers or dimers with other members of the same subfamily (94). The third domain, the LRR region, has been implicated in ligand sensing and autoregulation of not only NLRs but TLRs (93, 94). The LRR is formed by tandem repeats of a structural unit consisting of a β strand and an α helix and is composed of 20–30 amino acids that form a horse-shoe shaped structure rich in the hydrophobic amino acid leucine (220). The NLRPs LRR gene is made up of tandem repeats of exons of exactly 171 nucleotides, which encode one central LRR and two halves of the neighboring LRRs (221). This particular modular organization possibly allows extensive alternative splicing of the LRR region leading to maximum variability in the ligand-sensing unit (94). However, a recent publication by Tenthorey et al. (222) analyzing a panel of 43 chimeric NAIPs, showed that LRR was unnecessary for NAIP/NLRC4 inflammasome ligand specificity, leading them to propose a model in which NAIP activation is instead triggered by ligand binding to NACHT-associated helical domains. This recent evidence suggests that the ligand-sensing function of the LRR domain in NLRs, which has been supported primarily by analogy to the well-established ligand-sensing function of the LRR region in TLRs, needs to be re-examined.

The most widely studied NLRs during *H. pylori* infection are NOD1 and NOD2, which are expressed in epithelial and antigen-presenting cells, and are known to specifically recognize peptidoglycan-derived peptides (γ-d-glutamyl-meso-diaminopimelic acid and muramyl dipeptide, respectively). An early study, attempting to determine the mechanism whereby *H. pylori* delivers peptidoglycan to cytosolic host NOD1, demonstrated that *H. pylori* peptidoglycan is delivered to the host cell via a type IV secretion system (223). More recently, Hutton et al. (224) showed, for the first time, that cholesterol-rich microdomains called lipid rafts, were important for the type IV secretion system-dependent peptidoglycan delivery and subsequent NF-κB activation and IL-8 production, mediated by NOD1. Interestingly, Kaparakis et al. (225) reported a novel mechanism in Gram-negative bacteria, including *H. pylori*, for the delivery of peptidoglycan to cytosolic NOD1 in host cells that involves outer membrane vesicles that enter epithelial cells through lipid rafts. In addition, Necchi et al. (226) demonstrated the formation of a particle-rich cytoplasmic structure (PaCS) in *H. pylori*-infected human gastric epithelium having metaplastic or dysplastic foci, where VacA, CagA, urease, outer membrane proteins, NOD1 receptor, ubiquitin-activating enzyme E1, polyubiquitinated proteins, proteasome components, and potentially oncogenic proteins like SHP-2 and ERKs colocalized, inferring that this structure is likely to modulate inflammatory and proliferative responses during *H. pylori* infection.

The recent finding that NF-κB and AP-1 complexes can be physically translocated to the nucleus in response to NOD1 activation has led to the view that NOD1 is likely to be essential for the induction of both NF-κB and AP-1 activation during *H. pylori* infection (227). A number of studies have shown up-regulation of *NOD1* expression in diverse human cell lines challenged with *H. pylori* in a *cag* PAI-dependent manner (228–230). Further, *H. pylori cag* PAI-positive strains have recently been shown to activate the NOD1 pathway through two components of the IFN-γ signaling pathway, STAT1 and IRF1 (228). Similarly, expression of NOD2 was shown to significantly sensitize HEK293 cells to *H. pylori*-induced NF-κB activation in a *cag* PAI-dependent manner (231). Further, NOD2, but not NOD1, seems to be required for induction of pro-IL-1β and NLRP3 in *H. pylori*-infected DCs (232).

A limited number of studies have assessed the interaction between NLRPs and other inflammasome-associated molecules, and *H. pylori*. NLRPs represent the largest NLR subfamily (14 genes have been identified in humans) and are believed to be the scaffolding proteins of inflammasomes (221, 233). NLRPs interact and recruit the adaptor apoptosis-associated speck-like protein (ASC) via PYD-PYD interaction (94). ASC (also known as PYCARD), a key component required for inflammasome formation, is formed by an N-terminal PYD and a C-terminal CARD (234, 235). This interaction leads to the recruitment of caspase-1, an intracellular aspartate specific cysteine protease, which subsequently leads to the maturation and release of pro-inflammatory cytokines (236).

An early study by Tomita et al. (237) demonstrated that in *H. pylori* positive patients antral IL-18 mRNA expression was increased as compared with *H. pylori* negative patients, however, mature IL-18 protein and active caspase-1 were found to be present in both infected and non-infected gastric mucosa. Interestingly, in the following year, Potthoff et al. (238) reported activation of caspase-3, -8, and -9, but not caspase-1, in AGS cells challenged with *H. pylori*. However, this finding is in contrast with subsequent studies, which have demonstrated an important role for NLRPs and inflammasome-related molecules in *H. pylori* infection. For example, Basak et al. (96) demonstrated that *H. pylori* LPS could activate caspase-1 through Rac1/PAK1 signaling, and that activated caspase-1 played a role in LPS-induced IL-1β maturation (96). Further, ASC-deficient mice challenged with *H. pylori* have been shown to exhibit higher bacterial loads and significantly lower levels of gastritis, when compared with wild-type mice, and were incapable of producing IL-1β or IL-18 and produced less INF-γ in response to *H. pylori* infection (239). Later, Hitzler et al. (240) showed in both cultured DCs and *in vivo* that *H. pylori* infection activates caspase-1, leading to IL-1β/IL-18 processing and secretion. Consistently, three studies, using human GC cell lines, gastric tissue, and murine models, confirmed increased expression of caspase-1, IL-1β, and IL-18 in *H. pylori*-infected cells (171, 241, 242). Further, Jiang et al. (243), also using a murine model, have reported the expression of NLRP3 inflammasome-related molecules as well as serum IL-1β, IL-18, and IL-33 levels to be significantly increased in *H. pylori*-infected mice. More recently, a study by Kim et al. (232) has shown that secretion of IL-1β by DCs infected with *H. pylori* requires TLR2, NOD2, and the NLRP3 inflammasome.

Given that little is known about the role of NLRPs, inflammasomes, or other molecules involved in the NLR signaling pathways in response to *H. pylori* infection, we recently assessed the gene expression of 84 different molecules involved in the NLR signaling pathways, through quantitative real-time PCR, using THP-1-derived macrophages infected with two strains of *H. pylori*, GC026 (GC) and 26695 (gastritis) (173). Our gene expression analyses showed five genes encoding NLRs to be significantly regulated in *H. pylori*-challenged cells (*NLRC4, NLRC5, NLRP9, NLRP12*, and *NLRX1*) (173). Interestingly, *NLRP12* and *NLRX1*, two known NF-κB negative regulators, were markedly down-regulated, while *NFKB1* and several NF-κB target genes encoding pro-inflammatory cytokines (*IFNB1, IL12A, IL-12B, IL6*, and *TNF*), chemokines (*CXCL1, CXCL2*, and *CCL5*) and molecules involved in carcinogenesis (*PTGS2* and *BIRC3*) were markedly up-regulated, in THP-1 cells infected with a highly virulent *H. pylori* strain isolated from a GC patient. These findings highlight the relevance of the NLR signaling pathways in gastric carcinogenesis and its close interaction with NF-κB (173).

Overall, current evidence clearly shows that, in response to *H. pylori*, members of the NOD and NLRP subfamilies are critical for generating mature pro-inflammatory cytokines/chemokines that are crucial for Th1 responses and lead to *H. pylori*-related gastric disorders.

### NOD-like receptors and gastric carcinogenesis

The role of the NLR signaling pathways in the biological continuum that characterizes GC remains relatively unexplored as a very limited number of studies have addressed this issue. For example, Allison et al. (228) have shown that NOD1 expression was significantly increased in human gastric biopsies displaying severe gastritis, when compared with those without gastritis, as well as in gastric tumor tissues, as compared with paired non-tumor tissues. In contrast, Jee et al. (244), who analyzed human GC tissues and GC cell lines, showed that a significant decrease in the expression of caspase-1 was associated with poor survival and was inversely correlated with p53 expression.

Given the reported interaction of *H. pylori* with NLRs and the importance of this in the development of gastric inflammation and subsequent carcinogenesis, as well as the production of DAMPs during tumor formation (245), further comprehensive studies of the functional relevance of NLRs activation during chronic gastritis, atrophic gastritis, intestinal metaplasia, dysplasia, and GC are clearly warranted.

### Genetic polymorphisms involved in the NOD-like receptor signaling pathway and gastric cancer

The majority of studies examining the association between polymorphisms involved in the NLR signaling pathways and the risk of GC have focused on *NOD1* and *NOD2* polymorphisms. Studies, conducted in a number of populations, have investigated the association between the polymorphisms *NOD1* rs2907749 (246), *NOD1* rs7789045 (246), *NOD1* rs2075820 (E266K) (179, 247), *NOD1* rs5743336 (180), *NOD2* rs7205423 (246), *NOD2* rs7202124 (164), *NOD2* rs2111235 (164), *NOD2* rs5743289 (164), *NOD2* rs2066842 (P268S) (248, 249), *NOD2* rs2066844 (R702W) (250), *NOD2* rs2066845 (G908R) (184), and *NOD2* rs2066847 (L1007insC) (184, 250), and risk of gastric precancerous lesions and GC (Table 3). Further, a recent meta-analysis by Liu et al. (251) that included six case–control studies has shown consistent associations between *NOD2* R702W, G908R, and L1007insC, and risk of GC.

**Table 3:** **Genetic polymorphisms in the NOD-like receptor signalling pathway that have been studied in relation to gastric precancerous lesions and gastric cancer**.

Reference	Journal	Population	Gastric lesion	Study sample size	Polymorphism	OR (95% CI)^a^
Castaño-Rodríguez et al. (173)	PLoS One	Chinese	GC	310	*CARD8* rs11672725	4.80 (1.39–16.58)
					*CARD8* rs10405717	2.46 (1.04–5.84)^b^
					*CARD8* rs2043211	0.19 (0.058–0.63)^b^
					*NLRP3* rs12079994	4.15 (1.70–10.12)^b^
					*NLRP3* rs3806265	3.33 (1.09–10.13)^b^
					*NLRP3* rs4612666	4.03 (1.15–14.16)^b^
					*NLRP12* rs2866112	4.73 (2.06–10.88)^b^
					*NLRP12* rs4419163	2.42 (1.12–5.23)^b^
					*NLRX1* rs10790286	4.00 (1.66–9.61)^b^
					*CASP1* rs2282659	4.65 (1.67–12.95)^b^
					*CASP1* rs530537	4.65 (1.67–12.95)^b^
					*CASP1* rs61751523	4.56 (1.57–13.28)^b^
Companioni et al. (164)	International Journal of Cancer	Caucasian	GC	1649	*NOD2* rs7202124	0.74 (0.61–0.89)
					*NOD2* rs2111235	0.77 (0.64–0.93)
					*NOD2* rs5743289	3.76 (1.33–10.63)^c^
Kim et al. (179)	Helicobacter	Korean	IM	412	*NOD1* rs2075820 (E266K)	1.0 (0.74–1.34)^d^
Wang et al. (246)	World Journal of Gastroenterology	Chinese	GC	456	*NOD1* rs2907749	0.50 (0.26–0.95)
					*NOD1* rs7789045	2.14 (1.20–3.82)
					*NOD2* rs7205423	0.82 (0.39–1.72)
Kupcinskas et al. (180)	BMC Medical Genetics	Caucasian	GC	574	*NOD1* rs5743336	1.01 (0.48–2.16)
			CG, AG and IM			0.78 (0.40–1.49)
Rigoli et al. (184)	Anti-cancer Research	Caucasian	CG	147	*NOD2* G908R	5.18 (1.65–16.09)
					*NOD2* L1007insC	3.66 (1.13–11.80)
Kara et al. (247)	Clinical and Experimental Medicine	Turkish	AG	150	*NOD1* rs2075820 (E266K)	13.35 (5.12–34.82)
			IM			2.71 (1.26–5.80)
Hnatyszyn et al. (248)	Experimental and Molecular Pathology	Caucasian	CG, AG, IM and GC	244	*NOD2* rs2066842 (P268S)	2.2 (1.40–3.30)
Angeletti et al. (250)	Human Immunology	Caucasian	GC	326	*NOD2* rs2066844 (R702W)	4.1 (1.75–9.42)^d^
					*NOD2* rs2066845 (G908R)	0.56 (0.17–1.65)^d^
					*NOD2* rs2066847 (L1007insC)	16.10 (3.83–67.81)^d^
Wex et al. (249)	Anti-cancer Research	Caucasian	GC	324	*NOD2* rs2066842 (P268S)	1.5 (1.05–2.17)
					*NOD2* rs2066844 (R702W)	1.3 (0.66–2.55)
Hofner et al. (189)	Helicobacter	Caucasian	CG	211	*NOD1* rs2075820 (E266K)	1.06 (0.66–1.73)

*GC, gastric cancer; IM, intestinal metaplasia; AG, atrophic gastritis; GC, chronic gastritis; OR, odds ratio; CI, confidence intervals*.

*^a^OR and 95% CI correspond to allele or genotype analysis, depending on available information in the article*.

*^b^Only in *H. pylori*-infected individuals*.

*^c^Significant only in non-cardia *H. pylori* CagA negative individuals*.

*^d^Results obtained through a Fisher’s exact probability test (two-tailed *P*-values) conducted in the current review using the information provided in the original article*.

Given the documented relevance of other NLRs in *H. pylori* infection and related GC, and that polymorphisms in genes such as *NLRP3* (252–255) and *CARD8* (255, 256) have been associated with inflammatory gastrointestinal disorders, we addressed, for the first time, the association between 51 polymorphisms in six genes (*NLRP3, NLRP12, NLRX1, CASP1, ASC*, and *CARD8*) involved in the NLR signaling pathways and risk of GC in a high risk Chinese population (173). In this study, we found novel associations between *CARD8* rs11672725 and the risk of GC, and *NLRP12* rs2866112 and the risk of *H. pylori* infection (Table 3). Further, we showed that the concomitant presence of polymorphisms involved in the NLR signaling pathways (*CARD8, NLRP3, CASP1*, and *NLRP12*) and *H. pylori* infection dramatically increased the risk of GC in Chinese (Table 3) (173).

The functional relevance of a number of these polymorphisms has been examined. For example, the introduction of *NOD2* R702W, a polymorphism located in the LRR of NOD2, into the HEK293 cell line, resulted in abrogation of *H. pylori*-induced activation of NF-κB signaling (231). Further, Maeda et al. (257) observed increased NF-κB activation in response to muramyl dipeptide in mice harboring a *NOD2* mutation that is homologous to *NOD2* rs5743293 (3020insC) in humans. However, it is worth noting that the conclusions described by Maeda et al. (257) must be interpreted with care given that the authors subsequently found a duplication of the 3’ end of the wild-type *Nod2* locus, including exon 11, which was targeted by the mutation, and therefore, they are currently working to recreate a mutant strain without such a duplication.

Given that investigation of the role of polymorphisms involved in the NLR signaling pathways in GC is a relatively recent field of research, further studies are required to assess the association between these polymorphisms and GC in a range of human populations, especially those at high risk of GC.

### Other pattern-recognition receptors and *Helicobacter pylori*-related gastric cancer

A further two PRR subfamilies, RLRs and CLRs, have been studied in relation to *H. pylori* infection and gastric carcinogenesis. It is well known that RLRs (RIG-I, MDA-5, and LGP2) induce type I IFN in response to different RNA viruses, however, investigation on the role of RIG-I-like receptors in the recognition of RNA derived from intracellular bacteria is very limited. Interestingly, a study by Rad et al. (112), which used mice lacking simultaneously up to four different TLRs, apart from identifying TLR2 and TLR9 to be important *H. pylori* recognizing PRRs, also showed that *H. pylori* 5′-triphosphorylated RNA can be sensed by RIG-I and can contribute to the TLR-independent type I IFN response to this bacteria in DCs. Further, Tatsuta et al. (258) have recently shown that MDA-5 expression was significantly increased in the human gastric antral mucosa of *H. pylori*-infected individuals. In addition, these authors showed that increased MDA-5 levels correlated with atrophy and intestinal metaplasia in the corpus of these individuals (258).

C-type lectin receptors bind to carbohydrates (mannose- or fucose-containing glycans) present on pathogens to tailor immune responses to viruses, bacteria, and fungi. DC-specific intercellular adhesion molecule-3-grabbing non-integrin (DC-SIGN) is a CLR expressed on the surface of both macrophages and DCs. Interestingly, it has been shown that *H. pylori* harbors fucosylated ligands that can be recognized by DC-SIGN (259). Further, *H. pylori* DC-SIGN ligands appear to actively dissociate the signaling complex down-stream of DC-SIGN (KSR1–CNK–Raf-1) to suppress pro-inflammatory cytokine production (259). In addition, *H. pylori* LPS Lewis blood-group antigens can bind to DC-SIGN in a fucose or galactose-dependent manner (260, 261) and this interaction appears to inhibit a Th1 response in DCs (262). It has also been demonstrated that *H. pylori*-induced IL-10 production in monocyte-derived DCs is significantly suppressed by the addition of anti-DC-SIGN, TLR2, or TLR4 antibodies, either alone or in combination, before *H. pylori* stimulation (263). Further, *in vitro* and *in vivo* experiments have shown that the expression of DC-SIGN is significantly higher in *H. pylori*-infected individuals as compared with that in their uninfected counterparts (264, 265).

To date, no studies have been conducted to determine the association between genetic polymorphisms involved in the RLR and CLR signaling pathways and GC, however, Kutikhin and Yuzhalin (266) have comprehensively analyzed the oncogenic potential of both RLRs and CLRs, suggesting that future oncogenomic investigations should focus on polymorphisms in *MRC1* (rs1926736, rs2478577, rs2437257, and rs691005), *CD209* (rs2287886, rs735239, rs4804803, and rs735240), *CLEC7A* (rs16910526), and *RIG-I* (rs36055726, rs11795404, and rs10813831).

Given the limited but consistent current evidence suggesting a role of RLRs and CLRs in *H. pylori* infection, and the documented interaction between these signaling pathways and other important PRRs in GC such as TLRs (267, 268) and NLRs (269, 270), further studies assessing the implications of RLRs and CLRs in *H. pylori*-related inflammation and subsequent carcinogenesis need to be conducted.

## Pattern-Recognition Receptors as Therapeutics Targets in Gastric Cancer

Pattern-recognition receptors are increasingly recognized as important players in immunotherapy as PRRs-specific agonists elicit a potent immune response to cancers, allergic diseases, and chronic viral infections, while reducing the risk of an uncontrolled and detrimental systemic inflammatory response (for comprehensive information on this topic refer to the reviews by Hedayat et al. (271) and Paul-Clark et al. (272).

In the context of gastric carcinogenesis, Tye et al. (132), using a GC murine model (gp130^F/F^) displaying elevated gastric TLR2 expression levels, have elegantly shown that genetic and antibody-mediated therapeutic targeting of TLR2 leads to a substantial reduction in stomach size and overall tumor burden, including the number of gastric tumors. A further example is presented in the study by Gradisar et al. (273), which suggested that MD-2 is one of the important targets of curcumin (diferuloylmethane), the main component of the spice turmeric (*Curcuma longa*) that is widely used for gastric disorders in the Indian subcontinent, in its suppression of the innate immune response to bacterial infection. Furthermore, curcumin was recently shown to polarize myeloid-derived suppressor cells, extracted from a human GC xenograft mouse model, toward a M1-like phenotype with an increased expression of CCR7 and decreased expression of the CLR dectin 1, being both observed *in vivo* (tumor tissue) and *in vitro* (splenic myeloid-derived suppressor cells from tumor-bearing mice) (274). In addition, a study by Yang et al. (171) demonstrated that the combination of catechins and sialic acid is effective in suppressing the inflammatory responses mediated by the inflammasome/caspase-1 signaling pathway in gastric epithelial cells during *H. pylori* infection. Also, poly(I:C), an agonist of TLR3 and RLRs, has been shown to have a pro-apoptotic effect *in vitro*, and has significantly inhibited xenograft growth of human GC in a mouse model, through up-regulation of RLRs (RIG-I, MDA-5, and LGP2) as well as an increased expression of Bcl-2 family members, suggesting that it may be a promising chemotherapeutic agent against GC (275).

Given that modulation of PRRs has been proven to be relevant in gastric carcinogenesis through diverse mechanisms, including suppression of *H. pylori*-induced inflammation and enhancement of cancer cell apoptosis, this approach should be considered a new and promising angle of immunotherapy in GC.

## Conclusion

**Figure 3:**
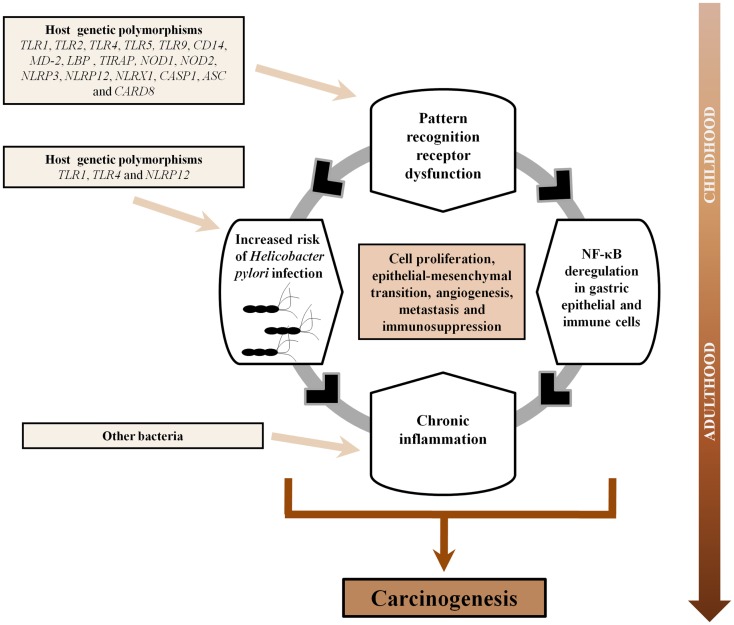
**Pattern-recognition receptors and gastric carcinogenesis**. Based on this comprehensive literature review, we propose a synergistic interaction between pattern-recognition receptors (PRRs) and *Helicobacter pylori* in gastric carcinogenesis. The association between PRRs and risk of GC might be a continuum commencing in childhood. Individuals harboring polymorphisms in PRRs could not only be more susceptible to acquisition of *H. pylori* in childhood but also would present deregulation of NF-κB in gastric epithelial and immune cells, and subsequent uncontrolled production of cytokines/chemokines, due to dysfunctional PRRs. This in turn would impact upon the direction and magnitude of the chronic inflammatory response to *H. pylori*. As *H. pylori*, the dominant bacterium in the stomach, gradually disappears upon the development of gastric atrophy, it is plausible that other microbial species might bloom in its absence and perpetuate local inflammation through further PRRs activation. Over time, the combination of these events would facilitate a number of features that promote gastric cancer development including cell proliferation, epithelial–mesenchymal transition, angiogenesis, metastasis, and immunosuppression.

In conclusion, abundant evidence supports the pivotal role of PRRs in gastric carcinogenesis as these receptors of the innate immune system, including TLRs, NLRs, CLRs, and RLRs, have been shown to recognize diverse components of *H. pylori*, the major risk factor of GC. In addition, PRRs are also involved in gastric carcinogenesis *per se* as these receptors are known to exert tumor-promoting functions (cell proliferation, epithelial–mesenchymal transition, angiogenesis, and metastasis) as well as immunosuppression during cancer. Given that host genetic variability in the TLR and NLR signaling pathways are known to be associated with an increased risk of *H. pylori* infection, the development of gastric precancerous lesions and GC, this knowledge has the potential to allow better prevention of GC through selective treatment and surveillance of individuals harboring high risk genetic profiles. Finally, given that PRRs are increasingly being used as a target for immunotherapy against both cancer and infectious diseases, the established relevance of PRRs in *H. pylori* infection and GC, could suggest that PRR agonists and/or antagonists may potentially improve the outcome of GC. Based on the extensive evidence presented in the current review, we propose a synergistic interaction between PRRs and *H. pylori*, which over time, could facilitate the sequence of events that characterizes GC development including inflammation, atrophy, intestinal metaplasia, dysplasia, and finally, GC (Figure 3).

## Conflict of Interest Statement

The authors declare that the research was conducted in the absence of any commercial or financial relationships that could be construed as a potential conflict of interest.
